# Explaining Firm Performance During the COVID-19 With fsQCA: The Role of Supply Network Complexity, Inventory Turns, and Geographic Dispersion

**DOI:** 10.1177/21582440231173671

**Published:** 2023-06-05

**Authors:** Weili Yin, Wenxue Ran

**Affiliations:** 1 Yunnan University of Finance and Economics, Kunming, China

**Keywords:** supply network complexity, geographical dispersion, inventory turns, firm performance, fsQCA

## Abstract

The COVID-19 pandemic has significantly affected firm performance. As a result, many studies have examined the significance of supply network complexity. Our paper uses the fuzzy set qualitative comparative analysis (fsQCA) method to investigate the causal relationships among the supply network complexity, geographic dispersion, inventory turns, and firm performance. Using a sample of 263 Chinese listed firms, we find that no single factor is necessary to achieve high firm performance during COVID-19 and reveal four paths to produce high performance: operational level driven, supply base complexity driven, and customer base complexity driven with supplier distance, and supply network complexity absence. Furthermore, our findings suggest that supply-based complexity-driven and customer-based complexity-driven can improve firm performance, but not all supply network complexity dimensions can improve firm performance. Hence, firms need to choose the suitable path based on their specific situations.

## Introduction

The competition in the 21st century is no longer between firms, but rather between supply chains (Christopher, 2000). With increased globalization, firms are more likely to reduce operating costs through global sourcing to improve their competitiveness (Sturgeon et al., 2009). With the sudden outbreak of the COVID-19 pandemic, the global supply chain is at risk of disruption. The firm performance was severely affected during the COVID-19 pandemic, therefore, firms need to increase their supply chain’s capacity to manage risk if they want to maintain performance in supply chain disruptions environment, and supply chain complexity is a critical measure to improve firms’ ability to cope with risk. Therefore, increasing the supply network complexity is a key strategy to deal with the supply chain disruption in the context of global supply chain disruptions and the COVID-19 pandemic. Supply chain disruption can be divided into upstream interruption and downstream disruption (Marley et al., 2014). Keeping adequate inventory and flexibility on hand is one of the most crucial ways to handle disruptions in the upstream supply chain. The number of customer bases can increase in a way in response to disruptions in the downstream supply chain. The increase in customer demand is crucial for customer-oriented firms. Increasing supply network complexity is an essential way to address supply chain disruption; increasing supply base complexity or customer base complexity are both ways to increase supply network complexity. However, previous research indicates that supply network complexity negatively affects firm performance, while some other research indicates that supply network complexity has a positive effect on firm performance.

Geographic dispersion is another critical factor influencing core firm performance (Lorentz et al., 2012). Geographic dispersion in the supply network is primarily measured by geographic distance (Lawson et al., 2019), which mainly includes the customer distance and supplier distance. The diversification of the supply base often requires more suppliers to support the regular operation of the core firm, particularly in the face of supply chain risks. Firms committed to reducing operating costs through global sourcing will increase the distance between the supplier and the core firm. The increased distance between suppliers and the core firm will also have a significant on the core firm’s ability to mitigate disruptions (Shao, 2013). Traditionally, increased customer distance is thought to have a negative impact on core firm performance, while, some studies show that core firms can achieve better financial performance even when their customers are far away.

The level of operating capacity can reflect the firm’s operation, and this study focuses on an important indicator of operating capacity: inventory turnover ratio (Hançerlioğulları et al., 2016) to investigate the relationship between inventory turnover rate and firm performance. Given the impact of inventory turnover on profitability and cash flow, there is also a significant impact of inventory turnover capacity on financial performance (S. F. Khatib et al., 2021). The operational capacity of the firm is a critical ability because a lower inventory level can reduce the inventory cost of the firm under regular operation (Sheffi & Rice, 2005), but when the demand fluctuates or the supply network is interrupted, this just in time (JIT) concept may not meet the firm’ regular operational needs. The relationship between inventory turnover and core firm performance has been discovered as an industry-specific feature in supply chain research (Eroglu & Hofer, 2011; MacDuffie et al., 1996). Although studies have shown a simple linear relationship between inventory turnover and firm performance (Koumanakos, 2008), few scholars have investigated the impact of the interaction between inventory levels and supply network complexity on financial performance improvement.

Given the paradoxical relationship between supply network complexity and firm performance, and few studies combine supply network complexity, geographic distance and inventory turnover to investigate the relationship between supply network complexity and firm performance. From a configuration perspective, we primarily employ the QCA method to investigate the causality asymmetry relationship between supply chain complexity, geographic dispersion, operational capacity and firm performance under the influence of COVID-19. QCA combines the advantages of qualitative and quantitative research to address the common causality complexity of social phenomena and identify conditional configurations with equivalent results (Charles, 2008), which can aid in our understanding of how antecedent conditions can be configured to produce the same results. Furthermore, QCA can identify the antecedent condition configurations that influence the presence of a given results, and investigate the equivalence between different antecedent configurations. The antecedent configurations for the presence or absence of a given results are analyzed separately. Besides, the antecedent conditions that lead to the same results may be multiple, and in many cases, not a single factor will lead to the results, so we examine how supply network complexity, geographic distance, and inventory levels interact to produce high firm performance from a configuration perspective. Therefore, the QCA method is primarily used in this study to investigate the relationship between supply network complexity and firm performance during the crisis. And, because China is the world’s largest manufacturing market and its supply network is more complex, we chose evidence from the Chinese market that best fits the characteristics of supply network complexity.

The contribution of this study to the supply chain field lies in the following aspects: First, this study reveals the asymmetrical and complex causal relationship between supply network complexity and firm performance. This research indicates that supply network complexity has a dual impact on firm performance. Our findings suggest that supply chain complexity is critical in improving firm performance during COVID-19, while in some cases, a lack of supply chain network complexity can actually improve firm performance during a crisis. Second, this study discovers that operational capacity (inventory turnover) is critical to a firm’s regular operation during COVID-19, and it also demonstrates that inventory turnover speed can help firms better manage risks and improve performance during the crisis. Third, we use configuration theory and fsQCA to investigate how supply network complexity, inventory level, and geographical dispersion can generate high firm performance. This also broadens the application of the QCA approach in the operations and supply chain field.

The remainder of this paper is organized as follows: Section 2 reviews the related literature; Section 3 is methodological aspects which include fsQCA methods, data sources, and measures and calibration; Section 4 discusses results analysis, which primarily includes the analysis of necessary conditions, the analysis of sufficient conditions, and the analysis of the resulting configuration path. Section 5 is the discussion and implications.

## Literature Review

COVID-19 had a significant impact on many firms around the world, particularly small and medium-sized enterprises (SMEs), many SMEs were unable to withstand the impact of the event and went out of business. The reason lies in that the supply chain disruption. Therefore, increasing the supply network complexity is critical for improving firm performance and viability. Furthermore, geographical diversification can mitigate the risk associated with a single supply, improving a firm’s ability to manage risk and improve performance, and a firm’s inventory level can also affect performance during COVID-19; the higher a firm’s inventory level, the better a firm’s ability to operate, and the more it can improve performance.

### Supply Chain Disruption Risk in the Context of COVID-19

The COVID-19 pandemic outbreak had a negative impact on global economic (Inoue et al., 2021; S. F. Khatib & Nour, 2021; Yu et al., 2021). The COVID-19 pandemic is devastating to the global economy and firm performance (Sarkis et al., 2020), and the COVID-19 pandemic represents a specific type of disruption risk (Sharma et al., 2021). COVID-19 differs from ordinary disruptions in three crucial ways: First, the long-term and unpredictable impact of the COVID-19 pandemic (T.-M. Choi, 2020). Second, unlike previous disruption, this new state raises new research concerns for supply chain organizations about how to manage long-term risks (T. Y. Choi et al., 2020). According to Charles Darwin, the species that can survive are not the strongest or the smartest, but rather the most adaptable to change (Darwin, 1909). Supply networks need constantly evolve and adapting to the ever-changing internal and external environment to survive in a radically changing environment. Third, the COVID-19 pandemic has significantly affected supply, demand, and logistics. The impact of the pandemic is not only forward-looking, but also has a backward impact, for example, disruptions to upstream supply chains can have ripple effects (Dolgui et al., 2018), and under the condition of the supply network disruption thoroughly, how to restore supply network and how to survive in the case of the severely disrupted is also a problem that must be solved (Ivanov, 2021).

The COVID-19 pandemic has had a negative impact on the entire society’s supply network, and firms must adopt the proper approach in the post-disruption stage to ensure supply chain survival (Paul et al., 2021). Some scholars argue that supply chain diversification is critical for improving firms’ resilience to crises and their ability to survive (Lin et al., 2021). Under the influence of the COVID-19 pandemic, more and more scholars are investigating how to use emerging technologies, such as blockchain technology, cloud computing, digital twin technologies, and so on, to improve the supply networks resilience (Sengupta et al., 2022). While many firms are actively deploying digital assets and expanding digital capabilities to deal with the negative impact of the COVID-19 pandemic on business operations. Firms must redesign themselves to respond to the impact of disruptions following the COVID-19 pandemic and to improve their ability to respond to risk (Dolgui et al., 2020; S. F. A. Khatib et al., 2022). Based on the resource-based theory, obtaining valuable, scarce, inimitable, and irreplaceable resources is critical for firms (Barney, 1996; S. F. A. Khatib et al., 2022), and firms lacking these resources will be unable to guarantee long-term survival during a crisis. Instead, it is necessary to organize and combine resources holistically, as well as investigate how to employ the firm’s resources in a way to increase its competitiveness (Sirmon et al., 2011). Firms should reorganize and integrates their resources holistically to address the situation of supply chain interruption and improve their capacity to meet consumer needs following the COVID-19 pandemic.

### Supply Network Complexity and Firm Performance Under Supply Chain Disruption Risk

Understanding the nature of the supply network complexity is another critical research area in supply chain. On the one hand, some studies show that supply network complexity can be positive, as it improves core firms’ ability to cope with market changes and competition; on the other hand, some studies show that supply network complexity can be negative, as managing complex supply networks often requires more financial and material costs (Wiedmer et al., 2021). As a result, there is currently no consensus on the relationship between supply network complexity and core firm performance. The current supply network complexity can be categorized in a variety of ways: based on the firm’s location and the position of the supplier network, it can be divided into upstream complexity, downstream complexity and internal complexity (Akın Ateş et al., 2022), external complexity and internal complexity (Blome et al., 2014).

Bozarth et al. (2009) define supply chain complexity as “the level of detailed and dynamic complexity shown by the products, processes, and relationships that make up the supply chain,” where detail complexity refers to the number of components that comprise a system, and dynamic complexity reflects the corresponding unpredictability of the system for a given set of inputs. Institutional control is a useful tool for controlling first-tier suppliers in complex multi-layer supply chains. Firms should cultivate specific adaptation mechanisms and strengthen the adaptability and self-organization (Najjar & Yasin, 2021). Furthermore, there is a correlation between supply network complexity and product selection, and developing appropriate methods can improve supply chain consistency by relating supply chain complexity (coordination, cooperation, and configuration) to product requirements and design characteristics (Blome et al., 2014). The increased supply network complexity has an impact on the core firm’s competitive position, and the traditional mapping method only shows the relationship between suppliers and customers in the vertical supply chain of the core firm. While the supply network map structure model broadens the perspective of suppliers and customers in the traditional supply chain to the complements and competitors in the horizontal supply chain (Kappel et al., 2020).

Supply chain complexity can also be conceptualized as both tangible and intangible complexity (Subramanian et al., 2015). According to empirical evidence, intangible supply chain complexity has a significant negative impact on firm performance (Pant et al., 2021). The supply network complexity can be strategically beneficial to the regular operation of the firm. As for how to respond to the supply network complexity, the firm can learn from the theoretical perspective of ambidexterity (exploration and exploitation) to respond to the supply network complexity, and provide practical support for practitioners to cope with necessary and unnecessary complexities (Turner et al., 2018). Blockchain technology can assist businesses in dealing with supply chain complexity while also reducing information asymmetry and opportunism (Najjar et al., 2023). Furthermore, previous research has demonstrated that board diversity and size do not affect on performance improvement (S. F. Khatib & Nour, 2021). The ability of the supply network to be resilient during a crisis is critical for firm’ survival in the current VUCA environment. And supply chain diversification is the key for building supply chain resilience. Diversification of the supply base is associated with higher profitability, whereas diversification of the customer base has higher demand during the crisis, but only shows higher profitability after the disruption (Lin et al., 2021). In the study of supply chain structure, supply chain density and geographical heterogeneity were found to be positively related to supply chain transparency, while supply chain aggregation was found to be negatively related to supply chain transparency (Gualandris et al., 2021).

By examining the supply network complexity types mentioned above, we classify the supply network complexity into upstream supply base complexity and downstream customer base complexity. In practice, it is more difficult for core firms to control the external complexity of the supply network than the internal complexity in the supply network environment.

### Inventory Turns and Firm Performance Under Supply Chain Disruption Risk

Complex supply base and customer base can increase operational complexity. To cope with theses complex situations, requiring not only additional investment in supply chain structure but also a higher level of coordination of these resources (Jacobs & Swink, 2011). Influenced by traditional JIT and lean thinking, many firms are committed to reducing inventory levels and advocating zero inventory, similar to Ford and Toyota, and other leading automakers. When supply networks are disrupted, supply networks need to improve supply chain resilience to mitigate the effects of the disruption (Roh et al., 2022). Preparing adequate redundancy is one of the most essential ways to restore supply chain resilience.

Inventory turnover is a crucial indicator for measuring inventory levels, which also represents a firm’s operational capacity (Vastag & Whybark, 2005). Effective inventory management in the supply chain is critical, and increasing inventory turnover is a crucial and challenging indicator for improving firms’ operational capacity. Firms improve their competitiveness by deploying and coordinating resources effectively while avoiding excess inventory (Demeter & Matyusz, 2011). Firms strive to reduce the supply and demand uncertainty to increase inventory turnover. Reduced uncertainty in supply and demand can help keep inventories at a safe level (Kadipasaoglu & Sridharan, 1995).

Many previous studies have taken inventory turnover as the antecedent variable and moderate variable and mediate variable to measure firm performance, and few studies have studied the relationship between inventory turnovers and firm performance from a configuration perspective. To achieve the required results, we will now move our attention to how inventory turnover interacts with supply base complexity and customer base complexity. Efficient inventory management requires effective integration and coordination within intra- and inter-organizational, and the expansion of the supply base and customer base requires additional coordination efforts. From the perspective of resource-based view, the core competitiveness of a firm is to have unique resources (Barney, 1996), and the number of suppliers owned by the firm and the number of customers obtained are among the firm’s resources, but the firm’s unique resources do not guarantee good performance. According to resource orchestration theory (Sirmon et al., 2011), how to effectively construct, bundle, and leverage firm resources by clearly articulating managers’ actions. Due to all competitive advantages are temporary, firms must arrange their resources to adopt competitive strategies and assist firms in achieving a series of competitive advantages over time. As a result, understanding how to organize inventory levels with supply base and customer base resources is essential for gaining a competitive advantage.

### Geographical Dispersion and Firm Performance Under Supply Chain Disruption Risk

Geographical dispersion of suppliers can bring certain advantages to core firms (Lorentz et al., 2012). By deploying multiple geographically dispersed suppliers, firms can partially mitigate the risks that arise during the procurement process and improve their capacity to deal with uncertainty. Globalization of production can improve firms’ access to markets, capabilities, and knowledge. By establishing stable relationships with multiple suppliers, firms can better collect market intelligence and attract outstanding talents (Wilson, 1995). Geographically dispersed suppliers may play a significant role in the firm’s overall supply network.

Customer geographical distance dispersion is important to improve the sales performance of firms (Handley & Benton, 2013). On the one hand, it can facilitate the realization of firm’s internationalization, while on the other, it can help firms obtain recognition in the local market and consumer support. Under the influence of the current pandemic, the risk of international and regional supply chain disruption increases. While the traditional view holds that geographical dispersion has a negative impact on countries’ bilateral trade. The geographical dispersion of upstream suppliers will increase warehousing and physical management costs for core firms (Creazza et al., 2010), while the dispersion of the downstream customer base will increase inventory costs and transportation costs, and the order fulfillment cycle. A higher average downstream customer transaction distance in the supply network will increase the risk of security in transit and performance risk. The increased average supply distance as a result of supply network decentralization will result in higher storage costs, inventory expenses, and supply times for the core firm, as well as more inventory in transportation.

Geographically dispersed supply base and customer base are increase the supply network’s complexity. As a result, the increase in complexity requires the coordination of activities along the supply chain. Especially under the impact of the pandemic, the supply base complexity, customer base complexity, and geographical distance increases will expand the impact of supply chain disruptions. The supplier base complexity, the customer base complexity, and the geographical distance may help the supply chain’s recovery from the disruption event during the recovery stage, thereby assisting in the restoration of firm performance.

**Figure 1.**
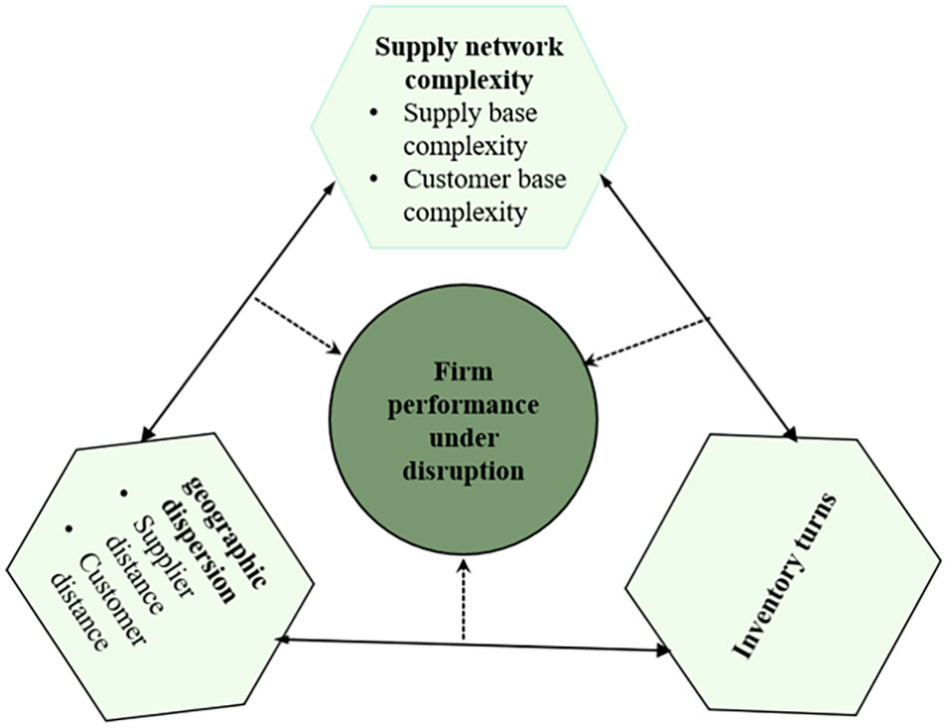
Research model.

In summary, this study uses configuration theory and fsQCA methods to examine the effects of supply network complexity (supply base complexity and customer base complexity), geographical dispersion (supplier distance and customer distance) and inventory turnover on firm performance under the influence of the COVID-19 from a holistic perspective. And exploring how supply network complexity, geographic dispersion, and inventory turnover are configured to produce high firm performance. Figure 1 depicts the conceptual model for this study.

## Methodology

### FsQCA Method

The qualitative comparative analysis focuses on configuration relationships that go beyond traditional linear relationships. Based on the research questions in this study, we chose the QCA approach for the reason that it has properties that allow us to discover the relationship between supply network complexity and firm performance under the influence of COVID-19. Traditional linear regression statistical approaches do not reveal this configuration link (Fiss, 2007; Meyer et al., 1993). The following are the advantages of the QCA method over traditional regression methods: (1) QCA focuses on multiple concurrent relationships between multiple antecedent conditions (Ragin, 2009). QCA uses a configuration perspective to conduct comparative analysis across cases, which can revealg the complexity of which antecedent conditions are configured to cause the expected results to occur or not to occur. (2) The purpose of the QCA method is to identify the antecedent conditions that produce the same results (Ragin, 2009), that is, all roads lead to Rome, and there will be multiple configurations between different antecedent conditions, and these configurations may lead to the same results. (3) The QCA method infers a causal relationship between the antecedent condition and the results through a set relationship rather than a correlation relationship (Rihoux & Ragin, 2008). By applying the perspective of set theory, the QCA method can identify the core and peripheral conditions for producing results, and the result-guiding practice based on the set relationship is more practical than the results obtained by using the net effect of the traditional regression method. QCA is classified into three types based on the data type: csQCA, mvQCA, and fsQCA (Ragin, 2009). Given the advantages of fsQCA in dealing with causal complexity methods, as well as the fact that it has evolved into a valuable and systematic approach to comparative social science research, and given the nature of the data in this study, we chose fsQCA to use as a research methodology for the study.

### Sample Selection and Data Sources

**Figure 2.**
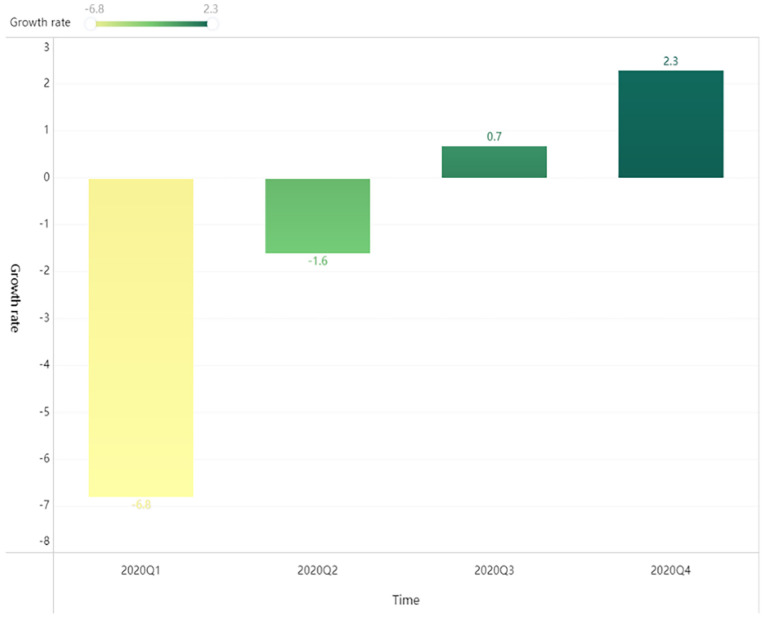
China’s GDP Growth year-on-year in 2020 (%).

**Figure 3.**
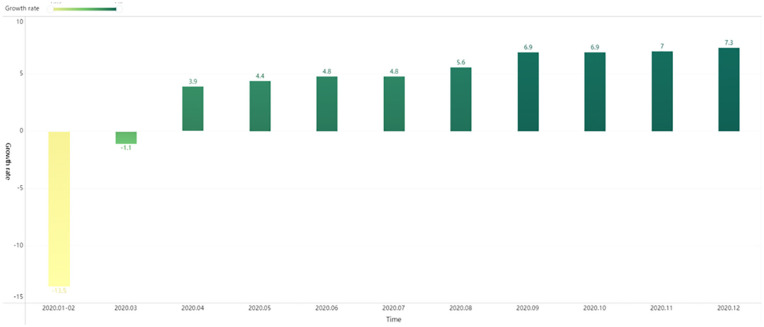
China’s GIP Growth year-on-year in 2020 (%).

We selected data from Chinese firms for two reasons: First, China is a major manufacturing country in the world which has a more complex supply network and customer base; Second, China has demonstrated a stronger recovery during COVID-19 and is better able to provide other countries or regions with some insight into improving firm performance during a crisis. And the data comes primarily from the CSMAR database (China Stock Market & Accounting Research Database), which is a research-oriented and accurate database in the economic and financial fields. We primarily use the CSMAR’s supply chain research database of Chinese-listed firms. Supplementary data from well-known domestic financial websites like Sina Finance, NetEase Finance, Flush, and Juchao Consulting. We primarily use the data in 2020 that the first quarter of 2020 is the most affected for firms, with the second to fourth quarters representing as the recovery period (As shown in Figures 2 and 3). The purpose of this study is to validate the relationship between supply network complexity and firm performance under COVID-19.

Based on the purchase and sales volume data provided by CSMAR data for the top five suppliers and customers, we only screened the data of the top five suppliers and customers in 2020, the data of suppliers and customers were matched to obtain2,808 pieces of firm data. Matching the geographic distance yields 268 pieces of data. And finally, we match the inventory turnover index with supply chain research database, we finally get 263 full data. The screening process of data in this study is shown in Table 1:

**Table 1.** Data Filtering Process.

Step	Database	Filter	Data obtained
1	CSMAR (Supply chain research database)	Year:2020; Match purchase and sales data	2808 data
2	Final data for step 1	Match with geographic distance	268 data
3	Final data for step 2	Match with inventory index	263 data

### Measurement and Calibration

#### Measurement of Results and Antecedent Conditions

Firm performance: The primary objective of business operations has always been to maximize a company’s profitability. In this study, we primarily used ROS to measure the firm performance, ROS is defined as net income of a firm (operating profit) divided by total sales revenue (main business income), and the ROS indicator indicates the percentage of the firm’s sales revenue converted into firm profit. ROS can evaluate a firm’s ability to make profits through sales, and the ability to make profits through sales during COVID-19 is a very important indicator for a firm, indicating that the firm can maintain regular business activities to sustain the firm profits during the crisis, as well as that the firm has a strong ability to cope with the crisis. The higher the ROS indicator, the stronger the profitability of the firm (Lin et al., 2021), especially under the influence of the pandemic, the profitability of the firm can reflect its ability of the firm to deal with risks. As a result, in this study, we primarily used financial data for 2020 to examine the relationship between firm supply network complexity and firm profitability under the influence of COVID-19. This is because 2020 is the year most affected by the pandemic, and firms that can still make a profit during this year demonstrate that they are more capable of coping with risk.

Supply base complexity: Since qualitative data on the connection with suppliers is difficult to obtain, we represent supply base complexity in this study primarily by the number of suppliers. Supply base complexity is mainly measured using the number of suppliers. The number of top five suppliers is used in this study to measure supply base complexity. When the proportion of the top five suppliers is higher, the supply base complexity is lower; when the proportion of the top five suppliers is lower, the supply base complexity is higher. The purchase information of the top five suppliers is provided in the supply chain management research area of the CSMAR database; we use the proportion of the top five suppliers to measure the number of suppliers, if the top five suppliers have a higher proportion of purchase, it indicates that the core firms have fewer suppliers; if the proportion of purchase of the top five suppliers is low, it indicates that the firm purchases of suppliers are relatively dispersed, implying that the core firms have a large number of suppliers.

Customer base complexity: Customer base complexity refers to the number of customers a firm has and the complex relationships between customers that need to be managed. The study primarily employs the number of customers held by the core firm to indicate customer complexity, because qualitative data, such as how each customer interacts with the business, is challenging to quantify. While the CSMAR database provides information on the sales of the top five customers, we primarily use the proportion of the top five customers’ sales to measure the number of customers. If the proportion of sales of the top five customers is relatively high, it indicates that the core firm has fewer customers and a more concentrated customer base; on the other hand, if the proportion of sales of the top five customers is low, it indicates that the firm’s customers are more dispersed, that is, the core firm has a large number of customers.

Supplier distance: Supplier distance mainly refers to the distance between the supplier and the core firm. The greater the distance between the supplier and the core firm, the more time and effort will be required for the firm to organize the upstream and downstream links in supply chain interruption. The greater the distance between the supplier and the core firm, the more time and effort will be required for the firm to organize the upstream and downstream links in supply chain disruption. Geographic distance directly affects a firm’s delivery time and ability to respond to supply chain issues (Manuj & Mentzer, 2008). The geographical distance between suppliers and core firms is mostly used in this study to refer to the distance between suppliers. The majority of the data on supplier distances comes from the CSMAR supply chain research database.

Customer distance: Customer distance is the geographical distance between the core firm and the customer. The geographical proximity between the customer and the core firm is primarily reflected in customer distance. The customer distance data in this study is primarily derived from the supply chain research data in the CSMAR database, which uses the geographical distance between the customer and the core firm to refer to the customer distance, and the data provided by the CSMAR database provides data support for our research.

Inventory turns: Although different industries, business forms, and supply chain business households will eventually fall on inventory and capital, all supply chain operations revolve around inventory and capital movements (DeCampos et al., 2022). The number of times that inventory is turned over during f a specific period. The number of times that inventory is turned over during the course of a specific period. It is an indicator of how fast or slow inventory turnover is. A higher turnover rate indicates better sales. The source of inventory turnover data, we are mainly supported by the inventory turnover data disclosed by well-known financial websites in China: Sina Finance, NetEase Finance, etc.

#### Calibration of Results and Antecedent Conditions

QCA is a set theory method used to discover the causal relationship between conditions and results, which is used to reveal the complex relationship between conditions and results. Uncalibrated data is difficult to read and less useful, therefore, calibration is crucial for subsequent analytical processes. In comparison to the natural sciences, social science research generally lacks direct calibration criteria. And few QCA research directly connected to the results and conditions discussed in this study have yet to be reported, hence there are no external standards to refer to. To overcome the problem of lack of theory and experience in the calibration process, this study follows the method of calibration using objective quantile values in mainstream QCA research (J. N. Lee et al., 2019), that is, using results and conditions 90%, 50% and 10% quantiles represent anchor points that are full in, the crossover point and fully out respectively, and this calibration method is supported and used by many mainstreams studied calibration to representing (Delmas & Pekovic, 2018; Garcia-Castro & Francoeur, 2016; Greckhamer & Mossholder, 2011), the calibration anchor point of various antecedent conditions and results is shown in Table 2:

**Table 2.** The Set and its Calibration Points.

Sets	Fuzzy set calibration		
	Fully out	Crossover point	Fully in
Supplier distance	3.29	5.57	7.21
Customer distance	2.57	5.72	7.33
Supply base complexity	25.54	64.1	86.62
Customer base complexity	18.38	68.36	91.38
Inventory turns	1.10	4.97	28.97
ROS	−0.32	0.05	0.21

## Results Analysis

### Necessary Condition Analysis

FsQCA can identify the necessary and sufficient conditions to achieve results. Before beginning a fsQCA, it is critical to conduct the necessary examination of the antecedent conditions that cause the results. A necessary condition is a core condition that must be presence for a results to occur, and if this condition is absent, the result will not occur. Table 3 shows the results of the analysis of the necessity of the five antecedent conditions for achieving high performance in this study, and we can see that the consistency value of all the antecedents for the desired goals is less than 0.9 (Schneider & Wagemann, 2012). It indicates that no single condition is necessary for achieving high firm performance during COVID-19, implying that the influence of the configuration of antecedent conditions on high performance should be investigated through a truth table.

**Table 3.** Necessary Condition Analysis of Antecedents.

Antecedents	High performance	
	Consistency	Coverage
Supplier distance	0.672200	0.613534
~Supplier distance	0.626697	0.703858
Customer distance	0.648910	0.685721
~Customer distance	0.604278	0.705289
Customer base complexity	0.626697	0.661337
~Customer base complexity	0.597079	0.697948
Supplier base complexity	0.633416	0.669881
~Supplier base complexity	0.590087	0.688120
Inventory turns	0.615110	0.766969
~ Inventory turns	0.668038	0.667306

### Sufficiency Analysis of Conditional Configuration

When using fsQCA to determine the adequacy of the configuration for the antecedent conditions, we mainly follow the mainstream research principle: setting the appropriate number of cases, and according to the recommendations of the basic research, we set 1.5% of the number of cases as the frequency threshold of this study, because the number of cases in this study is 263, so this study sets the frequency threshold as 4. The consistency level of the configuration is set to 0.8, and the full name of PRI consistency is a proportional reduction in inconsistency, which is an alternative measure of subset relations, PRI consistency can be used to avoid a concurrency relationship between the result and the negative result of a configuration (i.e., a configuration causes the result to occur and can also lead to its result not occur). The recommended PRI consistencies are 0.75, 0.65, and 0.5, Greckhamer et al. (2018) noted that configurations with PRI scores less than 0.5 showed significant inconsistencies. Therefore, based on the data in this study, we chose 0.5 as the consistency threshold, manually changed the truth table with a PRI consistency below 0.5 as 0, and then ran a standardized analysis. fsQCA generates three solutions: complex solutions, intermediate solutions, and parsimonious solutions. Core conditions are those that exist in both intermediate and parsimonious solutions, and peripheral conditions are those that only appear in intermediate solutions Core conditions are necessary factors, it is critical to the achievement of results, and peripheral conditions play a supporting role, these conditions are less important than core conditions, but they exist in the intermediate solution.

Therefore, the fsQCA 3.0 software was used in this study to analyze the antecedent configurations leading to high performance under the influence of COVID-19, and these different configurations represent different combinations to achieve the same result. At the same time, according to the process of configuration theorization, the discovered configuration is named (Furnari et al., 2021). In this study, six antecedent configurations lead to high performance (as shown in Table 4), and configuration 1 to 3 can be summarized as one path. We can eventually synthesize four solutions about how supply network complexity is configured to generate high firm performance in COVID-19 by configuration theorization (Table 5).

**Table 4.** Configurations for Achieving High Performance in fsQCA.

	Configurations					
	1	2	3	4	5	6
Supplier distance				•	•	
Customer distance	⊗	⊗				•
Supplier base complexity		•				⊗
Customer base complexity			•		•	⊗
Inventory turns	•		•	•		⊗
Raw coverage	0.45	0.43	0.40	0.40	0.42	0.26
Unique coverage	0.06	0.08	0.01	0.03	0.08	0.04
Consistency	0.81	0.79	0.81	0.82	0.75	0.84
Solution coverage	0.87					
Solution consistency	0.72					

*Note*. • Core conditions presence; ⊗Core conditions absence; • Peripheral conditions presence; ⊗ Peripheral conditions absence.

**Table 5.** Configurations Named After Configuration Theory.

	Configurations					
	Operational level driven			Supply base complexity driven	Customer base complexity driven with supplier distance	Supply network complexity absence
Supplier distance			•		•	
Customer distance	⊗			⊗		•
Supplier base complexity				•		⊗
Customer base complexity		•			•	⊗
Inventory turns	•	•	•			⊗
Raw coverage	0.45	0.40	0.40	0.43	0.42	0.26
Unique coverage	0.06	0.01	0.03	0.08	0.08	0.04
Consistency	0.81	0.81	0.82	0.79	0.75	0.84
Solution coverage	0.87					
Solution consistency	0.72					

*Note*. • Core conditions presence; ⊗ Core conditions absence; • Peripheral conditions presence; ⊗ Peripheral conditions absence.

After configuration naming, this research discovered four paths to high performance under the effect of COVID-19. Its overall consistency is 0.72, which is greater than the consistency threshold of 0.7, indicating a high level of consistency in our results; and an overall solution coverage is 0.87, indicating that our resulting configurations can cover most cases. The first path is an operational level-driven path, with three sub-paths, the first sub-path is the inventory turnover rate as the core condition, and the absence of customer distance as a peripheral condition can achieve high performance; The second sub-path is that in the case of high inventory turnover and high customer complexity, firms can also achieve high performance; The third sub-path is high inventory turnover with a long distance from the supplier, the firm can achieve high performance. This path demonstrates that inventory turnover is a key indicator of a firm’s operational capabilities. The higher the firm’s inventory turnover rate, the stronger the firm’s operating capacity the stronger the realization ability of the firm’s inventory assets, and the faster the inventory turnover. The raw coverage of these three paths is 0.45, 0.40, and 0.40 respectively, these coverages is higher compared to several other paths, implying that many firms achieve high firm performance through these paths.

The second path is supply base complexity driven: where high supply base complexity is the core condition, and customer distance is absent as the core condition, high performance can be achieved. This path demonstrates that the supply base of the firm is relatively complex, indicating that the firm has a large number of suppliers. Firms with a large backup supply base can improve their ability to cope with risk. The raw coverage of this path is 0.43, which is quite high compared to several other paths, implying that many firms achieve high firm performance through this path.

The third path is customer base complexity driven by supplier distance: high firm performance can be achieved when high customer complexity is the core condition and the long distance of the supplier is the core condition. In the case of high customer complexity, it can be demonstrated that the firm has a large customer base, and a diversified customer base can help the firm buffer the impact of COVID-19 on its performance. The raw coverage of this path is 0.42, which is pretty high compared to several other paths, implying that many firms achieve high firm performance through this path.

The fourth path is supply network complexity absence: this path demonstrates that high firm performance can be achieved when customer distance is present as a core condition, supply complexity and customer complexity are absent as core conditions, and inventory turnover is absent as a peripheral condition. In this path, the representative firms are Covestro Technology and China General Nuclear Power, and this kind of firms are that are far away from customers, while supplier complexity and customer complexity are relatively simple, customers are relatively single, suppliers are relatively single, and inventory turnover rate is relatively low, such firms are mainly represented by the technology industry and the power industries. The raw coverage of this path is 0.26, which is lower than the raw coverage of several other paths, indicating that only a small number of firms use it to achieve excellent firm performance.

### Robustness Test

To ensure the robustness of the results, we adjust the case consistency value from 0.8 to 0.82, as suggested by Leppänen et al. (2019), and continue to examine the above configurations that lead to high firm performance under the influence of COVID-19. As shown in Table 6, the configuration of our results has not changed compared with Table 5. According to Greckhamer, Furnari, Fiss and Aguilera (Greckhamer et al., 2018) suggestions, the adjustment of the parameters does not result in substantial changes in the number, components, and consistency and coverage of the configurations, that is the results of the analysis can be considered reliable and our results are of good robustness.

**Table 6.** Configuration Results After Increasing the Level of Consistency.

	Configurations					
	Operational level driven			Supply base complexity driven	Customer base complexity driven with supplier distance	Supply network complexity absence
Supplier distance			•		•	
Customer distance	⊗			⊗		•
Supplier base complexity				•		⊗
Customer base complexity		•			•	⊗
Inventory turnover	•	•	•			⊗
Raw coverage	0.45	0.40	0.40	0.42	0.42	0.26
Unique coverage	0.06	0.01	0.03	0.08	0.08	0.04
Consistency	0.81	0.81	0.82	0.75	0.78	0.84
Solution coverage	0.87					
Solution consistency	0.72					

*Note*. • Core conditions exist; ⊗ Core conditions are missing; • Edge conditions exist; ⊗ Edge conditions are missing.

## Discussions and Implications

### Conclusions

We study the impact of supply network complexity on firm performance in response to the risk of supply chain disruption in the recovery phase under COVID-19′s influence in this study. In contrast to traditional research, this study employs the configuration perspective to highlight a complex causal relationship. The findings show that there are four paths to high performance under the influence of COVID-19.

Path 1 is operationally level driven, indicating that inventory level is curial for the firm performance during the COVID-19. Inventory turnover index reflects the level of inventory management of firms. For firms, the faster the inventory turnover speed, the lower the level of inventory occupation, the stronger the liquidity, and the faster the inventory into cash or accounts receivable. Therefore, improving the inventory turnover rate of firms can improve liquidity of firms. The lower the inventory turnover rate, the worse the firm’s liquidity. Assessments of inventory turnover are carried out to ascertain how well the firm’s supply chain and level of demand are operating. However, the inventory turnover days are not as low as possible, and shortening the inventory turnover days may adversely affect the regular operation of the firm. This path also shows how inventory turnover interacts with customer and supplier portfolios to achieve higher firm performance. The higher the inventory turnover rate, the higher the turnover rate of firm’s raw materials, semi-finished products, and finished product inventories. To achieve a higher turnover rate, the firm will tend to reduce the variability between processes. The inventory turnover rate of firms can also reflect the effective coordination efforts of scheduling, procurement, and delivery activities, enabling firms to better deploy internal resources with downstream needs. This path demonstrates that high inventory turnover, even increased customer complexity, or a long distance from suppliers can improve firm performance. Firm performance can improve in cases of high inventory turnover and long customer distance. Overall, under the influence of COVID-19, the inventory turnover rate reflects the regular operating capacity of a firm (DeCampos et al., 2022). Under the impact of the pandemic, firms are more vulnerable to supply chain disruptions. And to cope with the supply chain disruption crisis, firms usually respond to risks by increasing inventory and redundancy, improving supply chain resilience and supply chain viability (Ivanov, 2022). Especially for firms with sufficient reserve inventory and more choices during the pandemic, the inventory and redundance they have can alleviate the impact of the supply chain crisis on the regular operation of firms. This path also demonstrates that firm profitability can be improved by improving and managing inventory turnover and combining geographical distance and customer complexity. Through the coordination and management of internal and external resources that belong to the firm regular operations, increase inventory turnover within the core firm, and increase firm profitability throughout the pandemic.

Path 2 is supply base complexity driven, which means sufficient backup suppliers can help firms to cope with the risks and improve firm performance. This result is consistent with previous research: there is a positive relationship between supply network complexity and financial performance, and while supply network complexity can create operational challenges and thus increase costs, the financial benefits may offset the costs associated with complexity (Akın Ateş et al., 2022). It also shows that complexity can be operationally harmful but strategically beneficial (Turner et al., 2018). Having a large number of backup suppliers is also the embodiment of the core resources owned by the core firms. Being able to release good signals to customers under the influence of the epidemic is made possible by having a large number of suppliers. Signal theory was developed on the assumption that information asymmetry exists, and signals can be extremely beneficial to both firms and individuals (Erevelles et al., 2001). Especially under the influence of COVID-19, there is a serious information asymmetry between firms and consumers, and core firms can use their good relationships with many supply chains to increase consumer confidence, that is, firms have enough ability to cope with supply chain risks, and can also increase customers’ psychological accounts and improve customers’ ability to trust firms. For core firms, having a diversified supply base belongs to the category of prior control, and through the choice of a diversified supply base in advance, firms’ ability to withstand risks posed by the pandemic can be improved. Prior contract control has the potential to improve supplier innovation performance (Cui et al., 2023). As a means of forwarding control, diversified supplier selection requires prior consideration of management and coordination among partners, which can also help firms carefully identifying and selecting partners and reducing the negative impact of a diversified supply base.

Path 3 is customer base complexity driven, which means that a larger customer base can assist the firm in dealing with the demand disruption risks, and this path may be suited for the demand-driven firms. In the case of high customer complexity, high firm performance is possible even if the supplier is located farther away, demonstrating that the firm is driven by customer needs, and customer needs are crucial to the survival of the firm. Especially in the wake of the COVID-19 pandemic, firms are more inclined to control their supply chains through demand-driven models (Chi et al., 2020). The firms under this path mainly include: Suning Global, Zhongwang Software, and Tools, etc., these firms are customer driven. For these firms, meeting consumer needs is the most crucial factor. Customers’ requirements must be met during a crisis for firms that prioritize meeting them, and a broad customer base gives firms the motivation for long-term survival.

And path 4 is supply network complexity absence. In this path, supply network complexity is not the crucial condition to high performance. This path follows the same conclusion as previous studies that supply network complexity has a negative impact on manufacturing performance (Bozarth et al., 2009). From these cases, we can obtain that this path mainly includes the technology industry and the power industry, they mainly produce single products and have single suppliers and customers, allowing firm’s to quickly master customer requirements. In other words, despite the fact that various business sectors were somewhat impacted in the first quarter of 2020, with the accelerated resumption of work and production, firms take the initiative to carry out production and operations. The concentration of suppliers and customers can cause firms to focus on a single product, and meet the needs of customers quickly. In the year 2020, due to the impact of the COVID-19 pandemic and the different economic development of various regions, the power supply and demand situation in various places is different, and firms have ensured the firm by paying close attention to the changes in the power market situation in various provinces and regions, through the implementation of the power sales strategy, combined with the power market situation in various provinces and regions overall economic benefits. Therefore, under the condition that supplier complexity and customer complexity are relatively low, that is, the suppliers and customers are relatively concentrated, firms can achieve better performance even if customers are far away during COVID-19 in 2020.

Contrary to prior studies, all four paths indicate four different configurations for obtaining high firm performance, which can assist firms in understanding the complex link between supply network complexity and performance. Our findings demonstrate the importance of supply network complexity in enhancing firm performance in times of crisis, but they also demonstrate that this is not the only way to achieve high firm performance during crisis; and lack of supply network complexity can also result in high performance during crises. Furthermore, our study also reveals that the level of operations plays a crucial role in improving performance (Krasnikov & Jayachandran, 2008).

### Theoretical Implications

(1) This study first reveals the complicated and asymmetrical causal relationship between supply network complexity and firm performance from a configuration perspective. This research indicates that supply network complexity has a dual impact on firm performance. For some firms, supply network complexity is beneficial to the regular operation of the firm during disruption risks. For example, in a complex supply network, a diverse supplier base and customer base are required to establish flexible, resilient, and adaptable supply chains. Therefore, firms can deploy supply network complexity to maintain regular operations and improve the ability to manage disruption risks in the face of interruption events such as COVID-19. While, these technology and electric power industries have a relative single supply base and customer base, supply network complexity absence is the key to ensuring the regular operation during the crisis. This requires them to focus on understanding and perceiving the needs of their customers all the time
(2) Operational capacity (inventory turnover) is critical to firm’s regular operation during COVID-19. According to research, the higher the inventory turnover, the easier it is for businesses to achieve positive results. Firms can enhance inventory turnover to improve their operational skills during COVID-19. The core of supply chain management is inventory management, and inventory capacity can be a reliable indicator of a firm’s performance. Innovation can improve operations management, and there is a link between innovation performance and inventory turnover (H.-H. Lee et al., 2015). Unlike previous research that investigated the relationship between single inventory turnover and firm performance, this study examines how inventory turnover, supply network complexity, and geographical dispersion can be used to improve firm performance from a configuration standpoint. The basic linear relationship between traditional inventory turnover and firm performance is extended by the configuration perspective which investigate configuration relationship between many antecedent conditions interacting to promote firm performance.
(3) This study is also one of the few studies that employs qualitative comparative analysis methods in the field of supply chain management. This is also consistent with the international mainstream journals initiative for future research directions in logistics and supply chain management (Ketchen et al., 2022). The qualitative comparative analysis approach is a new method of integrating qualitative and quantitative analysis that has evolved in recent years and is widely used in a variety of fields, including supply chain management. The QCA approach’s emphasis on causal complexity corresponds to the fact that supply chain results are frequently caused by the interaction of several diverse variables (Lou et al., 2022). As a result, the application of the QCA approach to supply chain management is more in line with actual needs and can provide beneficial information to practitioners.

### Practical Implications

(1) This study discovered a complex relationship between supply network complexity and firm performance during COVID-19. Firms should choose whether to improve their ability to cope with risk through supply network complexity based on their specific circumstances. Firms should consider risk management strategies from the standpoint of supply network structure when dealing with supply network risks, and incorporating supply network structure into management decision-making processes may yield new insights. By taking proactive measures to manage supply network risks, managers can expand their supply networks and decrease the negative effects of supply network complexity.
(2) This study demonstrates to managers how to think about configuration to reduce the risk of supply network disruption and improve firm performance. High firm performance is not caused by a single factor; rather, it is improved by a number of factors working together. As a result, rather than increasing performance from a single point of view, managers should improve performance from the standpoint of the whole. Duality has both positive and negative consequences (Park et al., 2020). To reconcile the conflict between classic linear research and supply chain research, new research methods and perspective on supply chain research are required. As a result, actual firm operators must abandon old basic linear thinking, develop the firm’s regular operation strategy from an overall macro perspective, increase the firm’s ability to actively respond to risks, and improve the firm’s ability to recover from interruptions.

### Limitations and Future Research

There are some limitations to this study that should be addressed in the future: First, this paper investigates the relationship between supply network complexity, geographic dispersion, inventory turns, and firm performance during COVID-19, and future research can investigate how the antecedent conditions are configured to improve firm performance from a different perspective. Such as innovation, supply network resilience, and supply chain survival (Yin & Ran, 2021), besides, we also can investigate the impact of digital transformation (Elgazzar et al., 2022), firm governance (PeiZhi & Ramzan, 2020), and new technology (Porter et al., 2020; Yli-Huumo et al., 2016) on firm performance under COVID-19 or supply chain disruptions. Second, we primarily use the data from Chinese listed firms, which may not be appropriate for the SME firms, so we can conduct detailed studies on different types of firms. Third, this study used data from a statistical database as well as domestic well-known financial and economic data released by the network. Questionnaire data can go beyond the financial data of qualitative data when compared to qualitative questionnaire data. Therefore, qualitative survey data can be used to present alternative viewpoints and confirm the research’s findings in the future.
